# Stratifin Promotes Hepatocellular Carcinoma Progression by Modulating the Wnt/*β*-Catenin Pathway

**DOI:** 10.1155/2023/9731675

**Published:** 2023-08-08

**Authors:** Shan-Ping Ye, Hong-Xin Yu, Wei-Jie Lu, Jun-Fu Wang, Tai-Yuan Li, Jun Shi, Xiao-Ye Cheng

**Affiliations:** ^1^Department of General Surgery, The First Affiliated Hospital of Nanchang University, No. 17 Yongwaizheng Street, Nanchang, Jiangxi Province 330006, China; ^2^Department of Hematology, The First Affiliated Hospital of Nanchang University, No. 17 Yongwaizheng Street, Nanchang, Jiangxi Province 330006, China

## Abstract

Abnormal stratifin (SFN) expression is closely related to the progression of several human cancers, but the potential roles of SFN in hepatocellular carcinoma (HCC) remain largely unknown. In this study, we found that SFN was upregulated in HCC cell lines and tissues and was positively associated with tumor size, poor differentiation, Tumor Node Metastasis (TNM) stage, and vascular invasion. In addition, high expression levels of SFN were associated with poor overall survival and disease-free survival. Biologically, downregulation of SFN suppressed tumor cell proliferation, epithelial–mesenchymal transition (EMT), invasion, and migration in vitro and tumor growth in vivo. However, overexpression of SFN promoted cell proliferation, EMT, invasion, and migration in vitro and tumor growth in vivo. Mechanistically, overexpression of SFN activated the Wnt/*β*-catenin pathway by promoting Glycogen synthase kinase-3 beta (GSK-3*β*) phosphorylation, decreasing *β*-catenin phosphorylation, promoting *β*-catenin transport into the nucleus, and enhancing the expression of c-Myc, whereas depletion of SFN inhibited the Wnt/*β*-catenin pathway. In addition, TOPFlash/FOPFlash reporter assays showed that overexpression or downregulation of SFN obviously increased or decreased, respectively, the activity of the Wnt/*β*-catenin pathway. Our results indicated that SFN plays an important role in HCC, possibly providing a prognostic factor and therapeutic target for HCC.

## 1. Introduction

Liver cancer is the sixth most common cancer and the fourth leading cause of cancer-related death worldwide [1, 2]. Hepatocellular carcinoma (HCC) accounts for 75–85% of primary liver cancers, leading to nearly 745,000 patient deaths worldwide annually and constituting a serious threat to public health [2, 3]. The incidence of HCC is regionally heterogeneous, and approximately 85% of HCC cases are estimated to occur in developing or less developed countries, among which 72% occur in Asia and more than 50% occur in China, resulting in a heavy economic burden on China and Chinese society [2, 4]. Unfortunately, most HCC patients have advanced-stage disease when diagnosed, and the incidence-to-mortality ratio is close to 100% [5]. One important reason is the high recurrence and metastasis rates [6]. Epithelial–mesenchymal transition (EMT) is an important process in tumor progression [7]. Currently, an increasing number of studies have indicated that the abnormal expression of genes is closely correlated with the progression of HCC, but the specific functions of most of these genes are still unclear [8].

Stratifin (SFN) is a checkpoint protein of the cell cycle and a regulatory factor of nuclear mitosis [9]. SFN is closely related to many cellular processes, especially the regulation of the cell cycle and signal transduction [10]. Moreover, SFN is an important regulatory protein that has been proven to have an impact on inflammatory processes [11]. Overexpression of SFN has been reported in many human tumors, such as ovarian cancer [12], breast cancer [13], lung cancer [9], and HCC [14]. However, the downregulation of SFN has also been observed in gliomas [10], the nervous system [15], and the reproductive system [15]. Hu et al. reported that upregulation of SFN was associated with a worse prognosis in ovarian cancer [12]. Deng et al. indicated that SFN was an independent risk factor for prognosis in patients with glioma [10]. Recently, several studies reported that SFN was upregulated in HCC [14, 16, 17]. However, the biological roles of SFN in HCC remain largely unknown, and its function in the process of EMT is still unclear.

In this study, we explored the role of SFN in HCC and found that SFN was upregulated in HCC tissues and cell lines. The expression of SFN was positively associated with large tumor size, poor differentiation, advanced Tumor Node Metastasis (TNM) stage, vascular invasion, and poor prognosis in HCC. We also found that SFN promotes the proliferation, migration, invasion, EMT, and tumorigenesis of HCC cells. Mechanistically, SFN activates the Wnt/*β*-catenin signaling pathway. Our results indicate the role and potential mechanism of SFN in HCC, possibly providing a therapeutic target and prognostic marker for HCC. A preprint of this manuscript has previously been published on the Research Square online platform [18].

## 2. Materials and Methods

### 2.1. Patients, Clinical Samples, and Follow-Up

A total of 34 pairs of HCC tissues and adjacent nontumor tissues were obtained from HCC patients in the Department of General Surgery of the First Affiliated Hospital of Nanchang University. All of the patients underwent radical resection between October 2017 and January 2018. The fresh specimens were stored in liquid nitrogen. The clinical data were collected from electronic medical records, and the survival information was obtained by follow-up. None of the patients received any adjuvant therapy before surgery and signed a written informed consent form. Our study was conducted in compliance with the Declaration of Helsinki and was approved by the institutional review of The First Affiliated Hospital of Nanchang University.

### 2.2. Cell Lines and Cultures

The HCC cell lines MHCC-97H and MHCC-97L were obtained from the Liver Cancer Institute of Fudan University (Shanghai, China). The HCC cell lines Huh7, SMMC-7721, HepG2, and the normal liver cell line L02 were obtained from the Chinese Academy of Sciences (Shanghai, China). All cells were cultured in Dulbecco's modified Eagle's medium (DMEM, Gibco, USA) containing 10% fetal bovine serum (FBS, Gibco), 100 U/ml penicillin, and 100 *μ*g/ml streptomycin (Gibco). All cells were placed in a humidified incubator with 5% CO_2_ and at 37°C.

### 2.3. RNA Extraction and Quantitative Real-Time PCR

Total RNA was obtained from the cultured cells or fresh-frozen tissues by using TRIzol Reagent (Invitrogen, USA) and was reverse transcribed into cDNA by using the PrimeScript RT Reagent Kit with gDNA Eraser (Takara, Japan) by the manufacturer's instructions. Quantitative real-time polymerase chain reaction (RT–qPCR) was performed in a StepOnePlus™ Real-Time PCR System (Applied Biosystems, USA) by using the TB Green® *Premix Ex Taq*™ II (Tli RnaseH Plus) Kit (Takara, Japan). *β*-Actin was used as an internal control. All gene expression levels were calculated by the 2^−△△CT^ method. The primers used in the current study were as follows: SFN: forward primer: 5′-TGACGACAAGAAGCGCATCAT-3′, reverse primer: 5′-GTAGTGGAAGACGGAAAAGTTCA-3′.

### 2.4. Establishment of Overexpressing and Knockdown HCC Cells

The knockdown and ectopic expression lentiviruses for SFN and the corresponding control lentivirus were synthesized by HanBio (Shanghai, China). The full-length human SFN cDNA sequence was inserted into the pHBLV-U6-MCS-CMV-ZsGreen-PGK-PURO retroviral vector to generate the pHBLV-SFN vector (named SFN), and the empty vector was considered the negative control vector (named Vector). Two shRNA oligonucleotide sequences against SFN were inserted separately into the retroviral vector to generate the pHBLV-shSFN vectors (names shSFN#1 and shSFN#2). The shRNA sequences were as follows: shSFN#1, 5′-GACGACAAGAAGCGCATCATT-3′; and shSFN#2, 5′-GCTCTCAGTAGCCTATAAGAA-3′. The scramble shRNA sequence was inserted into the vector, and the resulting construct was considered the shRNA negative control vector (named shNC). Transfection of cell lines was performed according to the manufacturer's protocol. In brief, 1 × 10^5^ MHCC-97H or SMMC-7721 cells were seeded in 6-well plates in DMEM with 10% FBS. The cells were transfected when the cell confluence was approximately 60%. The medium was replaced with 1 ml of fresh medium with 20 *μ*l of lentivirus solution. After 4 hours, another 1 ml of fresh medium was added to each well. The medium was replaced after 24 hours. The stably transfected cell lines were selected by puromycin at a final concentration of 2 *μ*g/ml. The efficiency of transfection was verified by RT–qPCR and western blotting (WB).

### 2.5. Cell Proliferation Assay

Cell proliferation assays were conducted using a Cell Counting Kit-8 (CCK-8; Dojindo, Japan) according to the manufacturer's protocol. Briefly, 5 × 10^3^ MHCC-97H or SMMC-7721 cells were seeded into 96-well plates after stable transduction with lentiviral vectors for 48 hours, with 5 wells established per group. Then, at 0, 24, 48, 72, and 96 hours, 10 *μ*L CCK-8 reagent mixed with 100 *μ*L culture medium was added to each well, and the plates were incubated in an incubator for 2 hours. Finally, a multimode plate reader (TECAN SPARK 10M, Switzerland) was used to measure the absorbance at 450 nm.

### 2.6. Colony Formation Assay

Transduced cells (1000 cells/well in the SFN overexpressing group and 500 cells/well in the SFN knockdown group) were plated in 6-well plates, and then the medium was replaced every five days. After two weeks, the cells were fixed with 4% paraformaldehyde and stained with 1% crystal violet. Then, the 6-well plates were washed with running water. The cell colonies were counted in the images.

### 2.7. Wound Healing Assay

After transduction for 48 hours, MHCC-97H or SMMC-7721 cells were seeded into 6-well plates. When the cells were 95% confluence, a scratch wound was made with a 200 *μ*L pipette tip by drawing a line on the surface of the cells in each well of 6-well plates. Images of wound healing at 0 and 24 hours were photographed using a microscope (10×) in each well.

### 2.8. Migration and Invasion Assays (Transwell)

Transwell chambers (Corning, USA) containing a membrane with a pore size of 8 *μ*m, and 24-well plates were used to perform the cell migration and invasion assays. For the migration experiments, 5 × 10^4^ cells were seeded into the upper chambers in 200 *μ*L of DMEM without FBS, whereas 700 *μ*L of DMEM containing 20% FBS was added to the bottom plates. For the invasion experiments, 50 *μ*L Matrigel/DMEM (1 : 8, BD Biosciences, USA) was added into the upper transwell chambers and then incubated overnight in a 4°C refrigerator for the next experiment. The cells were seeded and maintained in the same culture medium and conditions used in the migration experiments, although the number of cells was different (1 × 10^5^ cells). After incubation for 24 hours, the cells in the lower chamber or on the lower surface of the membrane were fixed in 4% paraformaldehyde and stained with 0.1% crystal violet. Then, the cells or Matrigel on the upper surface of the membrane were removed with a cotton swab. HCC cells were counted in five random fields by using a microscope (10×).

### 2.9. The Xenograft Mouse Models

Six-week-old male BALB/c nude mice purchased from Hunan SJA Laboratory Animal Co., Ltd., were used to investigate the role of SFN in tumor growth (six mice for each group). A total of 5 × 10^6^ lentivirally transduced HCC cells in 100 *μ*l DMEM containing 50% Matrigel were subcutaneously injected into the right upper flank regions of the mice. The tumor length (*L*) and width (*W*) were measured every three days. The formula 1/2 × (*L* × *W*^2^) was used to calculate the tumor volume. After 34 days, the mice were placed into the euthanasia chamber, and carbon dioxide was injected into the chamber at a rate of 30% of the volume of the euthanasia chamber per minute. After the animals stopped breathing and their pupils dilated, the next experiments were carried out. The tumors were excised for further study. All animal studies were approved by the Medical Experimental Animal Care Commission of the First Affiliated Hospital of Nanchang University.

### 2.10. Luciferase Reporter Assay

The reporter plasmids encoding TOPFlash or FOPFlash with TCF/LEF DNA-binding sites and the control plasmid pTK-RL were purchased from Beyotime Biotechnology (China). Infected cells were plated in 24-well plates, and the pTK-RL and Flash plasmids were cotransfected into the cells. After 48 hours, firefly luciferase activity was measured and normalized to Renilla luciferase activity.

### 2.11. Western Blotting

Total protein from cells and tissues was extracted with Radio Immuno Precipitation Assay (RIPA) buffer containing protease inhibitors. After centrifugation, the supernatants were collected for subsequent experiments. NE-PER™ Nuclear and Cytoplasmic Extraction Reagents (Thermo Scientific, USA) were used to extract nuclear proteins. The BCA Protein Quantitation Kit (Pierce, USA) was used to measure the protein concentration. A total of 30 *μ*g of protein from cells or tissues was separated by 8–12% sodium dodecyl sulphate-polyacrylamide gel electrophoresis (SDS–PAGE) and transferred onto polyvinylidene fluoride (PVDF, Millipore, USA) membranes. The PVDF membranes were blocked with 5% skim milk for 1 hour at 37°C and incubated with primary antibodies against SFN (ab193667, 1 : 1000, Abcam), *β*-actin (ab8226, 1 : 1000, Abcam), E-cadherin (#3195, 1 : 1000, Cell Signaling Technology), N-cadherin (#13116, 1 : 1000, Cell Signaling Technology), vimentin (#5741, 1 : 1000, Cell Signaling Technology), Matrix metalloproteinase 2 (MMP-2) (ab92536, 1 : 1000, Abcam), Matrix metalloproteinase 9 (MMP-9) (ab76003, 1 : 1000, Abcam), *β*-catenin (#8480, 1 : 1000, Cell Signaling Technology), non-phospho (active)-*β*-catenin (#19807, 1 : 1000, Cell Signaling Technology), Glycogen synthase kinase-3 beta (GSK-3*β*) (#12456, 1 : 1000, Cell Signaling Technology), phospho-GSK-3*β* (Ser9) (#5558, 1 : 1000, Cell Signaling Technology), c-Myc (ab32072, 1 : 1000, Abcam), and Histone H3 (ab1791, Abcam) overnight at 4°C. After washing with Tris-buffered saline in Tween buffer, the membranes were incubated with horseradish peroxidase (HRP)-conjugated anti-rabbit (SA00001-2, 1 : 5000, Proteintech) or HRP-conjugated anti-mouse (SA00001-1, 1 : 5000, Proteintech) secondary antibodies at 37°C for 1 hour. Both the primary and secondary antibodies were diluted with Primary & Secondary Antibody Diluent Buffer (Boster Bio, China). The band intensities were quantified by ImageJ (National Institutes of Health, USA), and the specific details were described previously [19].

### 2.12. SFN Expression in Online Databases

The gene expression profiles of liver HCC (LIHC) obtained from the RNA-Seq HTSeq-FPKM platform were downloaded from The Cancer Genome Atlas (TCGA) via the GDC Data Portal (https://portal.gdc.cancer.gov/repository) and consisted of data for 374 HCC tissues and 50 adjacent nontumor tissues. The limma package (R) was used to analyze the expression of SFN in tumor and nontumor tissues [20].

Regarding Gene Expression Omnibus (GEO) datasets (https://www.ncbi.nlm.nih.gov/geo/), we downloaded ten microarray expression profiles (GSE: 14520, 25097, 45114, 45436, 55092, 57555, 60502, 76427, 77314, and 101728) containing data for HCC tissues and nontumor tissues. Except for the difference in SFN expression in GSE77314, which was directly calculated from the XLSX data downloaded from GEO datasets, the difference in SFN expression between tumor and nontumor tissues in the other GEO series was determined by the Limma package (R) [20].

In the Oncomine database (https://www.oncomine.org/), four studies were comparing SFN expression between HCC and normal samples. We comprehensively analyzed the data in these four studies with the following thresholds: *P* value ≤1 × 10^−4^, fold change ≥2, and gene rank in the top 10%.

Immunohistochemical images were used to assess the difference in SFN protein expression between HCC tumors and normal liver tissues in The Human Protein Atlas (HPA) database (https://www.proteinatlas.org/).

### 2.13. Prognostic Analysis Based on Online Data

To investigate the prognostic value of SFN gene expression in patients with HCC, we conducted survival analysis and univariate and multivariate analyses. Univariate and multivariate Cox regression analyses and overall survival (OS) analyses were performed by the “survival” and “survminer” packages (https://cran.r-project.org/web/packages/) based on the R language. Data downloaded from the Kaplan–Meier plotter database (http://kmplot.com/analysis/) were used for survival analysis [21]. The OS time was defined as the time from surgery to death from any cause. The disease-free survival (DFS) time was defined as the time from surgery to death from any cause or the first relapse. The disease-specific survival (DSS) time was defined as the time from surgery to death from HCC. The recurrence-free survival (RFS) time was defined as the time from surgery to death with evidence of recurrence or the first relapse. THE GraphPad Prism software (version 7) was used to plot survival curves.

### 2.14. Statistical Analysis

The statistical analyses of the data in this study were performed using the SPSS 22.0 software and the GraphPad Prism 7. The Mann–Whitney *U* test, Student's *t* test or chi-square test, and Spearman's rank analysis were performed according to the type of variable. A two-tailed *P* value of <0.05 was considered statistically significant.

## 3. Results

### 3.1. SFN Is Highly Expressed in Human HCC Cell Lines and Tissues

**Figure 1:**
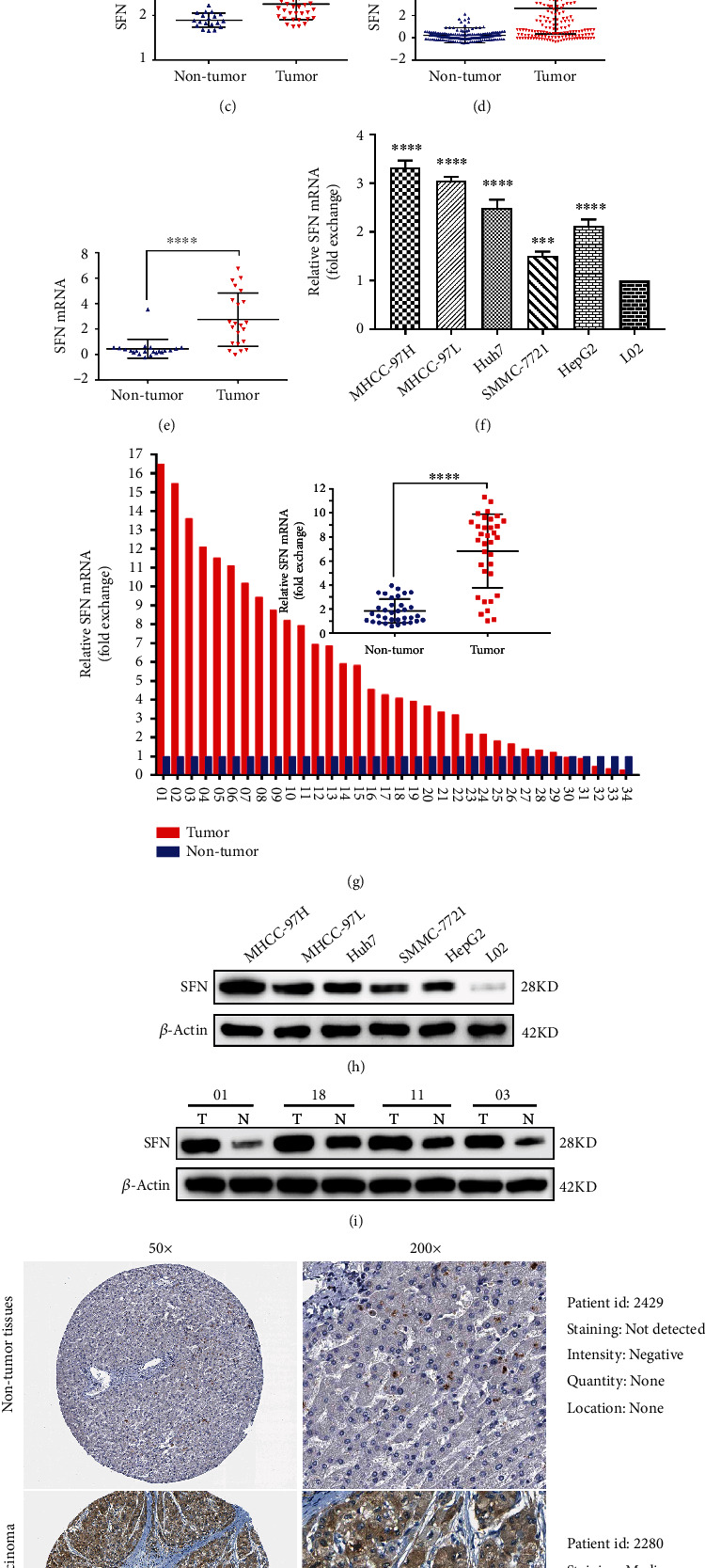
SFN gene expression is upregulated in HCC cells and tissues. (a) SFN mRNA expression in 377 HCC samples compared with 50 nontumor samples was analyzed in the TCGA database. (b, c, d, and e) SFN mRNA expression in HCC tissues in four Oncomine datasets (Roessler liver, Mas liver, Roessler liver 2, Wurmbach liver). (f) SFN mRNA expression was measured in five kinds of HCC cell lines and normal liver cells (L02) by RT‒qPCR in three independent experiments. (g) SFN mRNA expression was measured in 34 paired fresh HCC tissues and adjacent nontumor tissues by RT‒qPCR. (h) SFN protein expression was measured in five HCC cell lines and in normal liver cells (L02) by WB in three independent experiments. (i) Representative WB images from 34 paired fresh HCC tissues and adjacent nontumor tissues. (j) SFN protein expression in HCC tissues and nontumor tissues in the Human Protein Atlas. ∗∗*P* < 0.01, ∗∗∗*P* < 0.001, ∗∗∗∗*P* < 0.0001.

To determine the expression level of SFN in HCC, we first analyzed data from the TCGA database and found that the expression level of SFN in HCC tissues was higher than that in nontumor tissues (Figure 1(a)). Furthermore, we explored SFN expression in HCC in the Oncomine database. As shown in Figures 1(b), 1(c), 1(d), and 1(e), the SFN gene was overexpressed in HCC tissues in the Wurmbach Liver dataset, Mas Liver dataset, Roessler Liver 2 dataset, and Roessler Liver dataset compared with nontumor tissues. We further conducted a meta-analysis of the above four datasets in the Oncomine database and found that SFN was also overexpressed in HCC tissues compared with nontumor tissues (Supplement Figure 1A). In addition, we searched the GEO database and found that SFN expression was upregulated in ten GEO series compared with nontumor tissues (Supplement Figures 1B, 1C, 1D, 1E, 1F, 1G, 1H, 1I, and 1K).

To further confirm the level of SFN expression, we determined the expression level of SFN in HCC cell lines and normal L02 cells. As expected, both the mRNA and protein expression of SFN were upregulated in all HCC cell lines compared with L02 cells (Figures 1(f) and 1(h)). Furthermore, we examined the SFN mRNA and protein levels in fresh clinical HCC tissues. The HCC tissues had higher mRNA and protein expression levels of SFN than the adjacent nontumor tissues (Figures 1(g), 1(h), and 1(i)). Moreover, we explored the protein expression of SFN in the HPA based on immunohistochemistry. Consistent with the results of the clinical fresh HCC tissues from our center, SFN protein expression was also upregulated in HCC tissues in the HPA (Figure 1(j)). Based on these results, we concluded that SFN was significantly upregulated in human HCC cell lines and tissues.

### 3.2. SFN Was Closely Associated with Aggressive Clinicopathological Features and Poor Prognosis in HCC Patients

To further study the clinicopathological significance of SFN, we analyzed the correlation between SFN expression and clinicopathological characteristics in 34 HCC patients from our hospital. Patients were divided into high and low SFN expression groups based on the median expression level of SFN mRNA. SFN expression was associated with tumor size, degree of differentiation, TNM stage, and vascular invasion (*P* < 0.05; Table 1).

**Figure 2:**
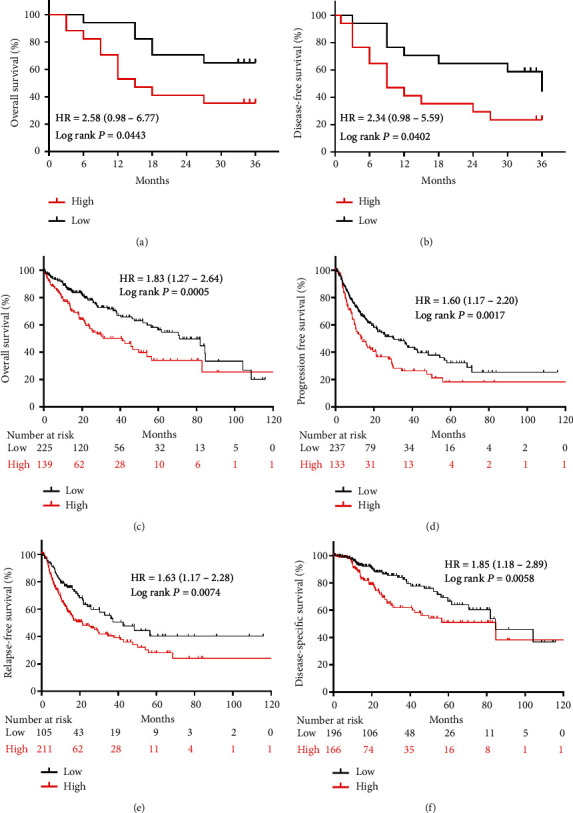
SFN gene expression is closely associated with poor prognosis. (a and b) The OS and DFS of HCC patients in the low (*n* = 17) and high (*n* = 17) SFN expression cohorts. (c–f) The OS, PFS, RFS, and DSS of patients with HCC in the Kaplan–Meier plotter online database.

To evaluate the prognostic value of SFN in HCC patients, we compared the differences in OS and DFS between 34 HCC patients in the high and low SFN groups. As expected, the high SFN group exhibited worse 3-year OS and DFS outcomes than the low SFN group (*P* < 0.05; Figures 2(a) and 2(b)). We further performed survival analysis using the Kaplan–Meier plotter online database. The results indicated that SFN overexpression in HCC tumors was significantly associated with worse OS, progression-free survival (PFS), relapse-free survival (RFS), and DFS in HCC patients (all log–rank *P* < 0.05; Figures 2(c), 2(d), 2(e), and 2(f)).

Furthermore, the clinicopathological data of 374 patients with HCC obtained from TCGA were used to evaluate the prognostic value of SFN in HCC by univariate and multivariate analyses. Due to the incompleteness of the clinicopathological data for many patients, we selected some common features (age, sex, tumor grade, and TNM stage) for further analysis and excluded unavailable data, including unknown, missing, Tx, Nx, and Mx data. Finally, 212 HCC patients were enrolled in this study. The univariate analysis indicated that TNM stage, T stage, M stage, and SFN expression level were significantly associated with OS (*P* = 6.71 × 10^−7^, 5.65 × 10^−7^, 0.0227, and 3.02 × 10^−6^, respectively, Table 2). As shown in Table 2, multivariate analysis indicated that SFN expression was an independent prognostic factor for OS (*P* = 0.0005).

In conclusion, our results suggested that SFN expression was closely associated with aggressive clinicopathological features and poor prognosis in HCC patients.

### 3.3. SFN Promotes HCC Cell Proliferation, Migration, and Invasion In Vitro and Tumor Growth In Vivo

**Figure 3:**
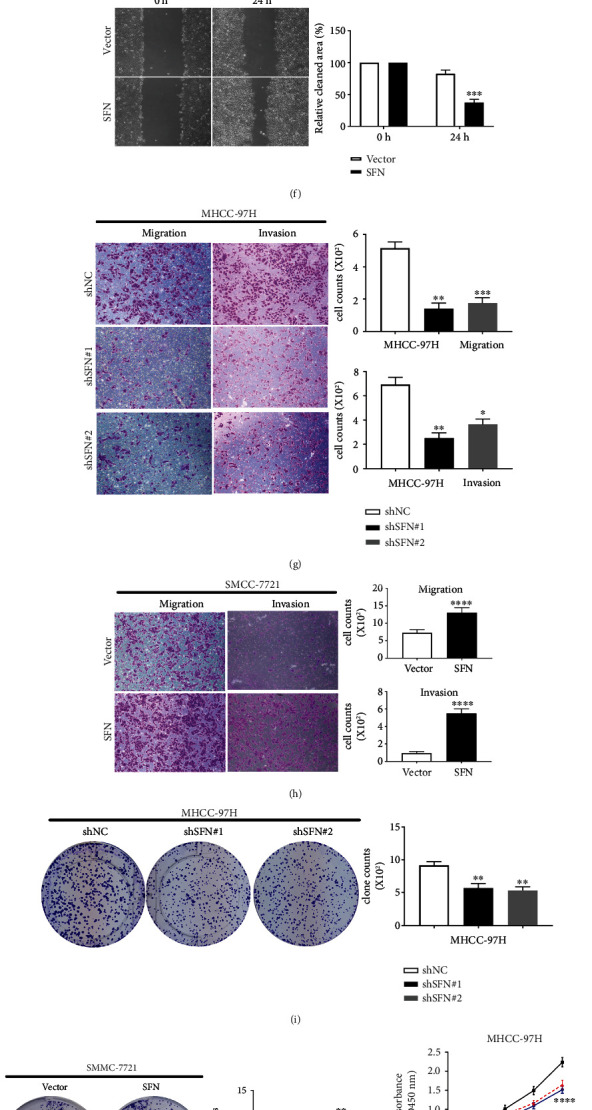
SFN promotes HCC cell proliferation, migration, and invasion in vitro and tumor growth in vivo. The efficiency of overexpression or knockdown of SFN was determined by RT–qPCR and WB in SMMC-7721 (a and b) and MHCC-97H (c and d) cell lines. Wound healing assays (e and f) and transwell migration assays (g and h) were used to explore the migratory ability of HCC cells with upregulated or downregulated SFN expression. (g and h) The invasion capacity of HCC cells with upregulated or downregulated SFN expression was examined by transwell invasion assays. (i and j) Colony formation assays were utilized to investigate the proliferation ability of HCC cells with altered SFN expression. (k and l) CCK-8 assays were used to evaluate the proliferation capacity of MHCC-97H cells with SFN knockdown and SMMC-7721 cells with SFN overexpression. (m) Xenograft mouse models were used to analyze the role of SFN in tumor growth. Images of subcutaneous tumors derived from MHCC-97H cells with SFN knockdown or SMMC-7721 cells with SFN overexpression in nude mice. (n and o) The weight and volume of the tumors in each xenografted mouse in the model. ∗*P* < 0.05, ∗∗*P* < 0.01, ∗∗∗*P* < 0.001, and ∗∗∗∗*P* < 0.0001. All data are from three independent experiments.

To explore the biological function of SFN in the progression of HCC, we upregulated SFN expression by lentivirus-mediated ectopic expression in SMMC-7721 (named SMMC-7721-Lv-SFN) cells, with the lowest SFN expression level, and we downregulated SFN expression by lentivirus-mediated knockdown in MHCC-97H (named MHCC-97H-Lv-shSFN) cells, with the highest SFN expression level. RT–qPCR and WB were used to estimate the knockdown efficiency (Figures 3(a), 3(b), 3(c), and 3(d)). To identify the function of SFN in the invasion and migration of HCC cells, we conducted transwell assays and wound-healing assays. In wound-healing experiments, we observed that the cells with SFN knockdown presented an obvious reduction in wound formation capacity compared with the control cells, whereas SFN overexpression enhanced the cell migration ability (Figures 3(e) and 3(f)). Similarly, the transwell assays showed that SFN depletion significantly decreased but SFN overexpression increased the migration and invasion abilities of HCC cells compared with control cells (Figures 3(g) and 3(h)). Furthermore, CCK-8 and colony formation assays were used to explore the proliferative capacity of two cell lines. We found that SFN knockdown inhibited colony formation and proliferation (Figures 3(i) and 3(k)). Conversely, overexpression of SFN promoted colony formation and proliferation (Figures 3(j) and 3(l)). Importantly, we evaluated the effect of SFN on tumor growth in vivo. In line with our results in vitro, we found that SMMC-7721-Lv-SFN cells exhibited an enhanced tumorigenic capacity compared with that of SMMC-7721-control cells. The MHCC-97H-Lv-shSFN cells exhibited a significantly reduced tumorigenic capacity (Figure 3(m)). The tumor weights in mice implanted with MHCC-97H-Lv-shSFN cells were significantly lower than those in mice implanted with MHCC-97H-control cells (Figure 3(n)), while the tumor weights in mice implanted with SMMC-7721-Lv-SFN cells were markedly higher than those in mice implanted with SMMC-7721-control cells (Figure 3(o)).

Taken together, these data demonstrated that SFN promoted HCC cell proliferation, migration, and invasion in vitro and tumor growth in vivo.

### 3.4. SFN Induces EMT and Activates Wnt/*β*-Catenin Signaling

**Figure 4:**
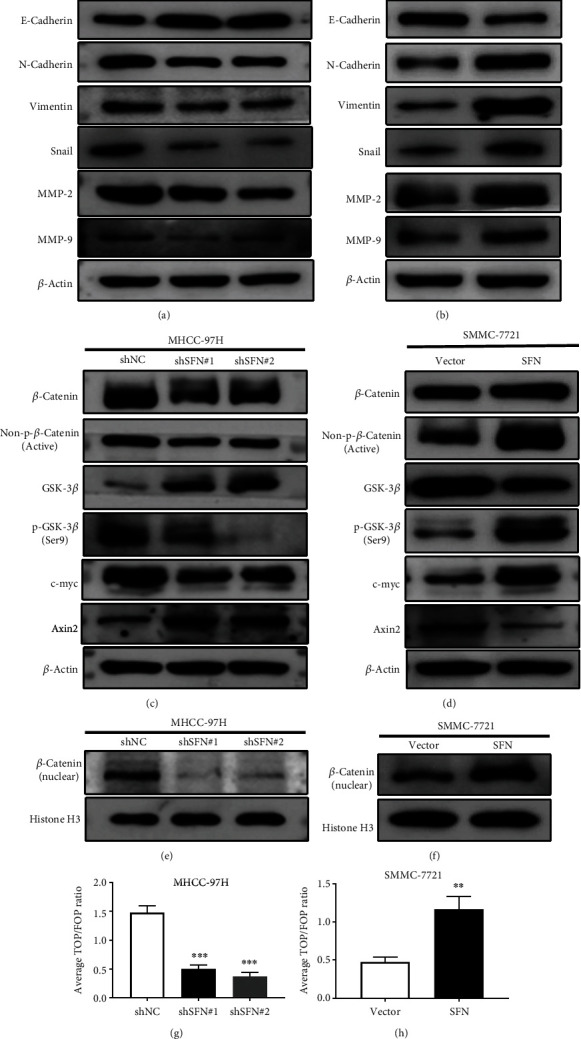
SFN induces EMT and activates Wnt/*β*-catenin signaling in HCC cells. (a) The protein levels of E-cadherin, N-cadherin, vimentin, Snail, MMP2, and MMP9 in MHCC-97H cells infected with lentiviruses expressing shSFN#1, shSFN#2, and shNC, as determined by WB. (b) The protein levels of E-cadherin, N-cadherin, vimentin, Snail, MMP2, and MMP9 in SMMC-7721 cells infected with lentiviruses carrying the SFN overexpression plasmid or Vector, as determined by WB. (c) The protein levels of *β*-catenin, non-phospho-*β*-catenin (active), GSK-3*β*, phospho-GSK-3*β* (Ser9), c-Myc, and Axin2 in MHCC-97H cells infected with lentiviruses expressing shSFN#1, shSFN#2, or shNC, as determined by WB. (d) The protein levels of *β*-catenin, non-phospho *β*-catenin (active), GSK-3*β*, phospho-GSK-3*β* (Ser9), c-Myc, and Axin2 in SMMC-7721 cells infected with lentiviruses carrying the SFN overexpression plasmid or Vector, as determined by WB. (e) The protein level of *β*-catenin in the nucleus of MHCC-97H cells infected with lentiviruses expressing shSFN#1, shSFN#2, or shNC, as determined by WB. (f) The protein levels of *β*-catenin in the nucleus of SMMC-7721 cells infected with lentiviruses carrying the SFN overexpression plasmid or Vector, as determined by WB. (g) TOP/FOP luciferase reporter activity in MHCC-97H cells with SFN knockdown. (h) TOP/FOP luciferase reporter activity in SMMC-7721 cells overexpressing SFN. All data are from three independent experiments.

EMT is an important process in tumor progression and is closely related to the processes of invasion and migration [22]. To further explore the mechanism of SFN in HCC progression, we examined the markers of EMT. We found that the ectopic expression of SFN in SMMC-7721 cells resulted in increased expression of N-cadherin and vimentin (mesenchymal markers) and decreased expression of E-cadherin (an epithelial marker), whereas SFN knockdown in MHCC-97H cells led to the opposite effects, as shown by WB (Figures 4(a) and 4(b)). In addition, we analyzed the changing trend in the expression of Snail, which is a key inducing transcription factor of EMT [23]. The results indicated that Snail is upregulated in SFN-overexpressing SMMC-7721 cells and downregulated in MHCC-97H cells upon SFN knockdown (Figures 4(a) and 4(b)). MMP2 and MMP9, members of the matrix metalloproteinase family, are closely associated with cell proliferation [24]. We evaluated MMP2 and MMP9 expression and found that MMP-2 and MMP-9 were upregulated in SFN-overexpressing cells and downregulated in SFN knockdown cells (Figures 4(a) and 4(b)).

Wnt/*β*-catenin signaling is a crucial pathway for HCC progression [25]. Therefore, we speculated that SFN may activate Wnt/*β*-catenin signaling to promote HCC progression. To verify this hypothesis, we explored the role of SFN in the expression of key markers of the Wnt/*β*-catenin signaling pathway, such as *β*-catenin and GSK-3*β* [25, 26]. *β*-Catenin is a key member of the Wnt/*β*-catenin signaling pathway and plays an important role in tumor progression [25]. In SFN-depleted MHCC-97H cells, we observed that *β*-catenin was downregulated (Figure 4(c)), whereas we observed that *β*-catenin was upregulated in SFN-overexpressing SMCC-7721 cells (Figure 4(d)). The active form of *β*-catenin is non-p-*β*-catenin, and its protein level reflects the activity of the signaling pathway [26]. The results from WB analysis indicated that downregulation of SFN could decrease the level of non-p-*β*-catenin protein in MHCC-97H cells (Figure 4(c)), whereas overexpression of SFN could increase the level of non-p-*β*-catenin protein in SMCC-7721 cells (Figure 4(d)). GSK-3*β* is another important component of Wnt/*β*-catenin signaling, participating in the degradation of *β*-catenin [27]. The results indicated that downregulation of SFN enhanced the expression of GSK-3*β* (Figure 4(c)) and that overexpression of SFN decreased the level of GSK-3*β* (Figure 4(d)). Once GSK-3*β* is phosphorylated at Ser9, its function in the degradation of *β*-catenin is compromised [28]. As shown in Figures 4(c) and 4(d), downregulation of SFN decreased the level of p-GSK-3*β* (Ser9) protein, but overexpression of SFN showed the opposite effect. Translocation of *β*-catenin into the nucleus is the best biomarker for activation of Wnt/*β*-catenin signaling [29]. We also evaluated the protein expression levels of *β*-catenin in the nucleus and found that SFN overexpression promoted *β*-catenin accumulation in the nucleus and that SFN knockdown exerted the opposite effect (Figures 4(e) and 4(f)). Moreover, nuclear translocation of *β*-catenin enhanced the expression of c-Myc [30]. The data derived from WB analyses indicated that the changing trend in c-Myc expression was positively correlated with the nuclear transcription of *β*-catenin (Figures 4(c), 4(d), 4(e), and 4(f)). In addition, we found that AXIN2 expression was down-regulated in SFN-overexpressing cells and up-regulated in SFN-silenced cells (Figures 4(c) and 4(d)).

Additionally, we used a TOP/FOPFlash assay to further confirm the activity of Wnt/*β*-catenin signaling. The TOP/FOPFlash reporter assay indicated that downregulation or overexpression of SFN obviously enhanced or suppressed, respectively, the activity of Wnt/*β*-catenin signaling (Figures 4(g) and 4(h)). Taken together, the above results indicated that SFN induces EMT and activates Wnt/*β*-catenin signaling.

To support the hypothesis that SFN affects the biological behavior of HCC cells by activating Wnt/*β*-catenin signaling, we further verified this hypothesis by treating SFN-overexpressing cells (SMMC-7721-Lv-SFN) with the Wnt signaling pathway-specific antagonist XAV-939. As shown in Supplemental Figure 2A, XAV-939 significantly inhibited the activity of the Wnt signaling pathway. The results indicated that the change trends in the levels of EMT-related markers were reversed by XAV-939 treatment in SFN-overexpressing cells (Supplement Figure 2B). Moreover, we found that the effects of SFN overexpression on the cell migration and invasion abilities were also reversed by XAV-939 treatment in SFN-overexpressing cells (Supplement Figure 2C).

## 4. Discussion

In the current study, we found that the expression of SFN is increased in HCC and significantly related to aggressive clinicopathological features and worse survival of patients with HCC. Moreover, our results indicate that SFN plays important roles in HCC proliferation, migration, invasion, and tumor growth and induces EMT by activating Wnt/*β*-catenin signaling. Therefore, SFN may be a novel oncogene in HCC and is expected to be a therapeutic target and prognostic factor for HCC.

SFN is a member of the conserved 14-3-3 protein family and plays important roles in various biological processes [31]. Overexpression of SFN has been reported in human malignant tumors. SFN is also a prognostic factor for many malignant tumors, such as ovarian cancer [12], glioma [10], and gallbladder cancer [32]. In our study, SFN was upregulated in HCC patients and associated with aggressive clinicopathological features. Moreover, we observed that patients with high SFN expression had worse survival outcomes than those with low SFN expression. We further validated these results in public databases. The TCGA, GEO, and Oncomine databases have greatly promoted cancer research because they contain large amounts of gene expression data and clinical data [33–35]. The gene expression data in the GEO database showed higher expression of SFN in HCC than in nontumor tissues. Our results were consistent with those of previous studies [36, 37]. Moreover, we investigated the prognostic value of SFN expression by using the Kaplan–Meier plotter online database and found that SFN overexpression in HCC tumors was significantly associated with worse OS, PFS, RFS, and DSS outcomes in HCC patients. Our data suggested that SFN promoted a poor HCC prognosis. Previous studies have referred to the potential role of SFN in tumors [38, 39].

SFN is closely correlated with the cell cycle, apoptosis, signal transduction, and protein trafficking [40]. Our results of functional experiments demonstrated that overexpression of SFN promoted HCC cell proliferation, migration, and invasion, whereas SFN knockdown in HCC cells resulted in the opposite effects. Our study also revealed that SFN could enhance tumor growth in vivo. EMT is vital in cancer development and progression [22]. EMT helps tumor cells acquire a mesenchymal phenotype and migration and invasion abilities [22]. Our results indicated that depletion of SFN suppressed but overexpression of SFN promoted EMT. Our data further enhance the understanding of the biological role of SFN in HCC progression.

In the current study, we further showed that SFN facilitated HCC cell proliferation, migration, invasion, tumor growth, and EMT by activating Wnt/*β*-catenin signaling. Wnt/*β*-catenin signaling plays an important role in the progression of HCC [22]. Gsk-3*β* and *β*-catenin are key components of the Wnt/*β*-catenin signaling pathway [22, 27]. Stable *β*-catenin expression plays a key role in the Wnt/*β*-catenin signaling output [25, 26]. Our results showed that the protein levels of *β*-catenin, non-phospho (active)-*β*-catenin, and phospho-Gsk-3*β* (Ser9) in SFN-overexpressing cells were increased. Cells with SFN knockdown showed decreases in these levels. To further confirm the effect of SFN on the Wnt/*β*-catenin pathway, we examined the protein level of *β*-catenin in the nucleus and found that SFN overexpression promoted the accumulation of *β*-catenin in the nucleus and that SFN knockdown exhibited the opposite effect. c-Myc is a downstream molecule of the Wnt/*β*-catenin signaling pathway, and its expression increases when *β*-catenin is translocated into the nucleus [30]. Our results showed that the changing trend in c-Myc expression was positively correlated with the nuclear transcription of *β*-catenin (Figures 4(c), 4(d), 4(e), and 4(f)), which again showed that SFN can activate the WNT signaling pathway. Axin2 is an important scaffold protein in the canonical Wnt/*β*-catenin signaling pathway [41]. Our results showed that SFN overexpression and knockdown led to increased and decreased Axin2 expression, respectively. Moreover, we used the TOP/FOPFlash luciferase reporter assay to assess the activity of the Wnt/*β*-catenin signaling pathway. The results indicated that overexpression of SFN activates the Wnt/*β*-catenin signaling pathway, whereas knockdown of SFN inhibits the Wnt/*β*-catenin signaling pathway. In addition, we provided further support for the hypothesis that Wnt/*β*-catenin signaling is related to the mechanism, by which SFN promotes HCC progression. XAV-939, an inhibitor of Wnt/*β*-catenin signaling, was adopted to inhibit Wnt/*β*-catenin signaling in SFN-overexpressing cells [42]. Treatment with XAV-939 inhibited the activity of Wnt/*β*-catenin signaling in SFN-overexpressing cells (Supplement Figure 1A). Consistent with the inhibition of Wnt/*β*-catenin signaling activity, treatment with XAV-939 led to the obvious abolishment of the process of EMT and a decreased ability of migration and invasion in SFN-overexpressing cells. Taken together, these results suggest that SFN can promote the progression of HCC by activating the Wnt/*β*-catenin signaling pathway. A previous study indicated that SFN can interact with Gsk-3*β* and inhibit Gsk-3*β* activity [43]. Our results also showed that the Gsk-3*β* catalytic activity in SFN-transfected cells was significantly lower than that in nontransfected cells. In line with the inhibition of catalytic activity, the phospho-Gsk-3*β* level was increased in SFN-overexpressing cells. Gsk-3*β* inactivation is controlled by its phosphorylation by the destruction complex [43]. Once Gsk-3*β* is phosphorylated and dissociated from the multiprotein destruction complex, *β*-catenin is released [43]. Thus, SFN regulates Gsk-3*β* and then activates the Wnt/*β*-catenin signaling pathway to promote the progression of HCC, which is the possible main mechanism revealed by the current research. However, this is just a preliminary exploration of SFN in HCC, and a more in-depth mechanism needs to be further investigated, especially its impact on the inflammatory process.

## 5. Conclusions

In conclusion, SFN is overexpressed in HCC tissues and cell lines, and SFN expression is correlated with tumor size, differentiation, vascular invasion, and TNM stage. High expression of SFN was associated with worse survival outcomes. SFN promotes cell proliferation, migration, invasion, and tumor growth and may be an oncogene in HCC. Moreover, SFN induces EMT and activates the Wnt/*β*-catenin signaling pathway. The results of the current analysis may identify a prognostic factor and therapeutic target for HCC in the future.

## Figures and Tables

**Table 1:** Clinicopathological features of 34 HCC patients between SFN high and low expression cohorts.

Features	qRT-PCR		*P* value
	Low (*n* = 17)	High (*n* = 17)	
Gender, *n*			
Male	15	13	0.656
Female	2	4	
Age, years			
≤50	7	9	0.732
>50	10	8	
HBV infection, *n*			
Positive	16	15	1.000
Negative	1	2	
AFP, ng/ml			
≤400	7	12	0.166
>400	10	5	
Tumor number, *n*			
Solitary	16	15	1.000
Multiple	1	2	
Tumor size, cm			
≤5	14	6	**0.013∗**
>5	3	11	
Vascular invasion, *n*			
Yes	4	11	**0.037∗**
None	13	6	
Tumor differentiation, *n*			
Well/moderate	17	9	**0.003∗**
Poor	0	8	
Liver cirrhosis, *n*			0.708
Yes	13	11	
None	4	6	
AJCC TNM stage, *n*			
I/II	14	6	**0.013∗**
III/IV	3	11	

AFP: alpha-fetoprotein; AJCC: American Joint Committee on Cancer; HBV: hepatitis B virus; HCC: hepatocellular Carcinoma; SFN: stratifin. ∗*P* < 0.05. Fisher exact test was used in all analyses.

**Table 2:** Univariate and multivariate survival analyses of OS in HCC patients from online databases.

Variables	Univariate analysis		*P* value	Multivariate analysis		*P* value
	HR	95% CI		HR	95% CI	
Age	1.006	0.987–1.025	0.538	1.012	0.991–1.033	0.275
Gender	0.845	0.516–1.385	0.505	1.022	0.585–1.787	0.938
Grade	0.996	0.726–1.366	0.979	1.159	0.821–1.636	0.403
AJCC TNM	1.921	1.485–2.485	0.001	2.041	0.642–6.488	0.227
T (tumor)	1.844	1.451–2.344	0.001	0.937	0.320–2.745	0.906
N (node)	2.018	0.492–8.279	0.330	1.080	0.125–9.310	0.944
M (metastasis)	3.867	1.208–12.374	0.023	0.755	0.191–2.987	0.689
SFN expression	1.009	1.005–1.013	0.001	1.008	1.004–1.013	0.001

CI: confidence interval; HCC: hepatocellular carcinoma; HR: hazard ratio; OS: overall survival; SFN: stratifin.

## Data Availability

The gene expression profiles of LIHC obtained from the RNA-Seq HTSeq- FPKM platform were downloaded from TCGA via the GDC Data Portal (https://portal.gdc.cancer.gov/repository). The GSE datasets (GSE: 14520, 25097, 45114, 45436, 55092, 57555, 60502, 76427, 77314, and 101728) were downloaded from the GEO database (https://www.ncbi.nlm.nih.gov/geo/). The Oncomine database (https://www.oncomine.org/) was used for meta-analysis of SFN expression. The immunohistochemical images were obtained from the Human Protein Atlas (https://www.proteinatlas.org/). Access to the database can be obtained from the corresponding author on reasonable request.

## Supplementary Materials

Supplementary Materials Supplement Figure 1 SFN expression in the online databases. (A) SFN expression from the meta-analysis of four datasets from the Oncomine database (*P* < 0.05). (B–K) The expression of SFN in ten GEO series (GSE: 77314, 101728, 14520, 25097, 45114, 45436, 55092, 57555, 60502, and 76427). ∗*P* < 0.05, ∗∗*P* < 0.01, ∗∗∗*P* < 0.001, and ∗∗∗∗*P* < 0.0001.Supplement Figure 2 XAV-939 can inhibit the biological behavior of HCC cells induced by SFN. (A) TOP/FOP luciferase reporter activity in SMMC-7721-Lv-SFN cells treated with XAV-939 (*P* < 0.05). (B) The protein levels of E-cadherin, N-cadherin, and vimentin in SMMC-7721-Lv-SFN cells treated with XAV-939, as determined by WB. (C) The migration and invasion capacity of SMMC-7721-Lv-SFN cells treated with XAV-939 was examined by transwell invasion assays. ∗∗*P* < 0.01, ∗∗∗*P* < 0.001, and ∗∗∗∗*P* < 0.0001.
