# Supplementary figures and images for: Stratifin Promotes Hepatocellular Carcinoma Progression by Modulating the Wnt/β-Catenin Pathway
Source: Int J Genomics. 2023 Aug 8;2023:9731675. doi: 10.1155/2023/9731675 (PMC10427227; doi:10.1155/2023/9731675)

**Supplement Fig 1**


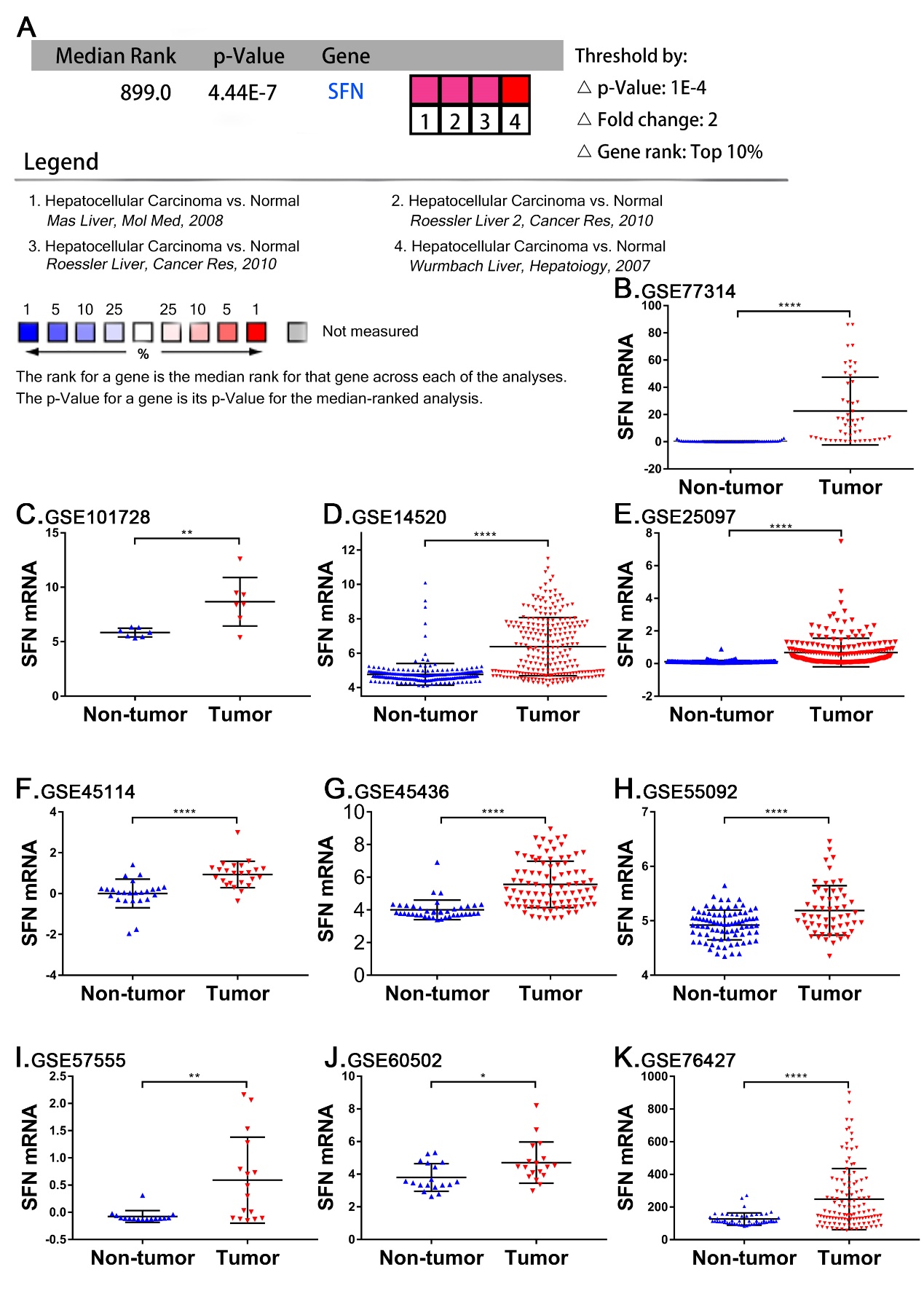


**Supplement Fig 2**


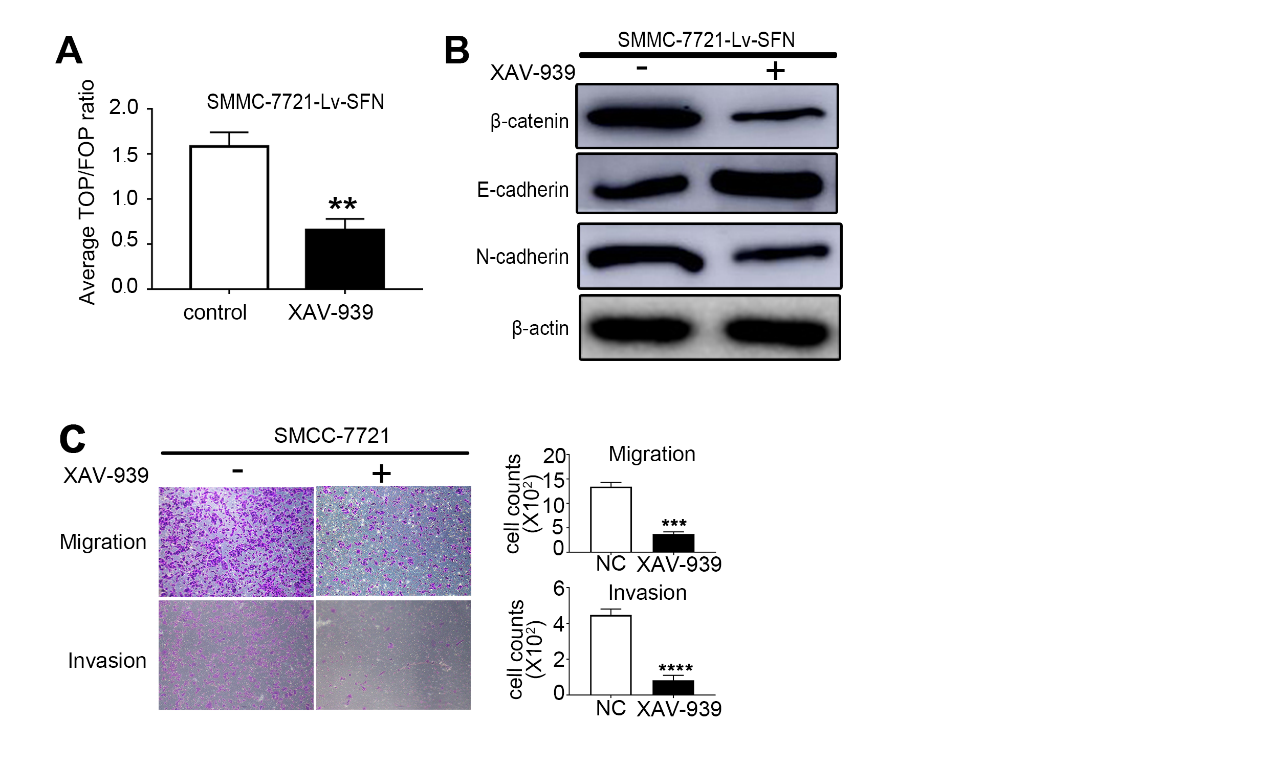

Supplement: Supplementary Materials — Supplement Figure 1 SFN expression in the online databases. (A) SFN expression from the meta-analysis of four datasets from the Oncomine database (P < 0.05). (B–K) The expression of SFN in ten GEO series (GSE: 77314, 101728, 14520, 25097, 45114, 45436, 55092, 57555, 60502, and 76427). ∗P < 0.05, ∗∗P < 0.01, ∗∗∗P < 0.001, and ∗∗∗∗P < 0.0001.Supplement Figure 2 XAV-939 can inhibit the biological behavior of HCC cells induced by SFN. (A) TOP/FOP luciferase reporter activity in SMMC-7721-Lv-SFN cells treated with XAV-939 (P < 0.05). (B) The protein levels of E-cadherin, N-cadherin, and vimentin in SMMC-7721-Lv-SFN cells treated with XAV-939, as determined by WB. (C) The migration and invasion capacity of SMMC-7721-Lv-SFN cells treated with XAV-939 was examined by transwell invasion assays. ∗∗P < 0.01, ∗∗∗P < 0.001, and ∗∗∗∗P < 0.0001. [file 9731675.f1.docx]
